# Perspiration after Sleep

**Published:** 1896-10

**Authors:** Frederic Preston


### PERSPIRATION AFTER SLEEP.

September 3d, 1896.

Dear Editor:—On page 359, August number of The Homœopathic Physician, Dr. Selfridge makes a very grave error in saying "there was only one remedy in the materia medica that had that symptom," viz.: "*After sleeping very profuse perspiration, which came on immediately on awakening.*"

Of course, you but publish the society proceedings; but it seems strange that among so many experts present at the time there was not one to challenge such a statement.

The following authorities will show that it was quite a serious error: See *Sambucus-niger*, *Guiding Symptoms*, Vol. IX, page 195; Allen's *Intermittent Fever*, second edition, page 219; Hering's *Condensed Materia Medica*, second edition, page 758; *Symptomen Codex*, Jahr, Vol. II, page 728.

Allen's *Encyclopædia of Pure Materia Medica*, Vol. VIII, page 480, says: "Profuse sweat without thirst *while awake from* 7 P. M. *to* 1 A. M.; drops stood on the face, and there was also perspiration all over; but after sleep he was more hot than sweaty, but without thirst." Again: "He was sweaty all over

*on awakening from sleep.* Two nights." Both symptoms are numbered from Hahnemann.

See C. Lippe's *Repertory,* page 259, first column: Perspiration less in sleep. Nux-v., Rumex., Sambucus.

See Bœnninghausen on *Intermittent Fever* (Kornderfer's translation), page 200: Sweat on awakening.

### FIFTY-TWO REMEDIES.

In first degree.—Ant-c., Paris q., Sambuc., Sepia, Sulphur.

In second degree.—Ars., Calc., Chel., Cinch., Clemat., Merc-v., Nux-v., Phos., Ran-bulb., Tarax.

The others, third degree.

This last, that of Bœnninghausen's, shows what an imperfect thing is C. Lippe's *Repertory.*

There are two headings in Bœnninghausen on *Intermittent Fever:*

First.—Sweat *when* awakening, of which I have given you remedies in first and second degree

Then there is "sweat after awakening."

Third degree.—Bell., Carb-an., Cinchon., Hep., Nux-v., Phos-ac., Sepia.

Second degree.—Bry., Phos.

First degree.—Sambucus.

I think that ought to settle it.

Now, I have myself *many times cured* intermittent fever on that symptom—"dry heat in sleep; profuse sweat on awakening"—with Sambucus. Where that symptom was present I have less frequently cured cases of the same disease with *Paris-quad.,* and once with Ant-crud. I, however, distinctly remember *one* epidemic of grippe where Samb. was very frequently necessary on account of the perspiration and other symptoms after sleep. I have this evening added Lac-can. to this symptom in all my repertories; but I know it would be quite wrong to omit all the others on anybody's dictum, though I have no doubt Dr. Selfridge knows much more materia medica than I do. I have sweated here in this vile sweat-box of a town over so many vile and varied cases of intermittent fever that a slip like that strikes me rather forcibly. Again, I long ago renamed *grippe* to suit myself. I call it "winter chills and fever," and as I have once been the victim for seven sempiternal weeks of the genuine summer article and four times of its winter "sosie" (double), I think I know at least how it feels, even if I don't know what it is.

Speaking of sweats, I have a four-page article by S. F. Shannon, M. D., of Denver, Col., pasted in my Bœnninghausen on *Intermittent Fever* entitled "Some Peculiar Sweats," and added to that is a note of my administration of "Gastein" (spring water, 6th dilution) to "a consumptive" in 1894 for "red sweat in axilla." When I say "red sweat" I mean that it looked red, and stained everything red that it touched (except the air). The sweat disappeared after the remedy was taken, and the "consumptive" is still above ground, coughing away.

Lest you might think me restrained by a certain false modesty, I make bold to state that I admire your contributor's article on "*warts*."* It is a good article, and though parts of it remind me in a vague way of the "*Contes Grassomillets*" of Monsieur Armand Sylvestre, I cannot think less of it for *that*. I fear that our theological wing will scarcely approve, however; but if it does not, why the little difference such disapproval could make would hardly rob us of the "sweet reasonableness" of its humor.

The more I look at my old copy of Hering's *Typhoid Fever* the more I think it is high time some one stepped into the breach and republished it.

Good-night. May angels of all denominations—Protestant, Catholic, Swedenborgian, Baptist, etc.—hover round your pillow, chasing the mosquitoes with their downy wings.

Yours,

FREDERIC PRESTON.

---

* See THE HOMŒOPATHIC PHYSICIAN for August, 1896, page 347.

